# Combined Cognitive-Motor Rehabilitation in Virtual Reality Improves Motor Outcomes in Chronic Stroke – A Pilot Study

**DOI:** 10.3389/fpsyg.2018.00854

**Published:** 2018-05-30

**Authors:** Ana L. Faria, Mónica S. Cameirão, Joana F. Couras, Joana R. O. Aguiar, Gabriel M. Costa, Sergi Bermúdez i Badia

**Affiliations:** ^1^Faculdade de Psicologia e de Ciências da Educação, Universidade de Coimbra, Coimbra, Portugal; ^2^Madeira Interactive Technologies Institute, Funchal, Portugal; ^3^Faculdade de Ciências Exatas e da Engenharia, Universidade da Madeira, Funchal, Portugal; ^4^CMM - Centros Médicos e de Reabilitação, Aveiro, Portugal

**Keywords:** virtual reality, stroke, motor rehabilitation, cognitive rehabilitation, task adaptation

## Abstract

Stroke is one of the most common causes of acquired disability, leaving numerous adults with cognitive and motor impairments, and affecting patients’ capability to live independently. Virtual Reality (VR) based methods for stroke rehabilitation have mainly focused on motor rehabilitation but there is increasing interest toward the integration of cognitive training for providing more effective solutions. Here we investigate the feasibility for stroke recovery of a virtual cognitive-motor task, the Reh@Task, which combines adapted arm reaching, and attention and memory training. 24 participants in the chronic stage of stroke, with cognitive and motor deficits, were allocated to one of two groups (VR, Control). Both groups were enrolled in conventional occupational therapy, which mostly involves motor training. Additionally, the VR group underwent training with the Reh@Task and the control group performed time-matched conventional occupational therapy. Motor and cognitive competences were assessed at baseline, end of treatment (1 month) and at a 1-month follow-up through the Montreal Cognitive Assessment, Single Letter Cancelation, Digit Cancelation, Bells Test, Fugl-Meyer Assessment Test, Chedoke Arm and Hand Activity Inventory, Modified Ashworth Scale, and Barthel Index. Our results show that both groups improved in motor function over time, but the Reh@Task group displayed significantly higher between-group outcomes in the arm subpart of the Fugl-Meyer Assessment Test. Improvements in cognitive function were significant and similar in both groups. Overall, these results are supportive of the viability of VR tools that combine motor and cognitive training, such as the Reh@Task. *Trial Registration:* This trial was not registered because it is a small clinical study that addresses the feasibility of a prototype device.

## Introduction

Stroke is one of the most common causes of adult disability and its prevalence is likely to increase with an aging population (WHO, 2015). It is estimated that 33–42% of stroke survivors require assistance for daily living activities 3–6 months post-stroke and 36% continue to be disabled 5 years later (Teasell et al., 2012). Loss of motor control and muscle strength of the upper extremity are the most prevalent deficits and are those that have a greater impact on functional capacity (Saposnik, 2016). Hence, its recovery is fundamental for minimizing long-term disability and improving quality of life. In fact, most rehabilitation interventions focus on facilitating recovery through motor learning principles (Kleim and Jones, 2008). However, learning engages also cognitive processes such as attention, memory and executive functioning, all of which may be affected by stroke (Cumming et al., 2013). Still, conventional rehabilitation methodologies are mostly motor focused, although 70% of patients experience some degree of cognitive decline (Gottesman and Hillis, 2010), which also affects their capability to live independently (Langhorne et al., 2011).

### What Is Missing in Conventional Cognitive and Motor Rehabilitation Methodologies?

Although motor and cognitive neurorehabilitation after acquired brain injury is strongly based on intensive training and task-specific learning for promoting neural reorganization and recovery (Alia et al., 2017; Galetto and Sacco, 2017), conventional methodologies still strive to accomplish this goal (Levin et al., 2014). Paper-and-pencil tasks are widely used in cognitive rehabilitation, and are assumed to be reliable and with adequate construct validity in the assessment and rehabilitation of cognitive functions after brain injury (Wilson, 1993). However, this methodology is not suited to deliver immediate feedback and reinforcement on progress, which is an important element to increase the motivation and avoid dropouts (Parsons, 2015). Additionally, when the dominant arm is affected by hemiparesis, performing paper-and-pencil tasks may become difficult or impossible. Regarding the motor domain, the persistent repetition of motor actions can be demotivating due to its repetitiveness and, because it is laborious and demanding in terms of human resources, it is not as intensive as it should be (Langhorne et al., 2009). In addition, the relationship between cognitive and motor deficits is increasingly being unveiled and cognitive effort appears to contribute to motor recovery (Pichierri et al., 2011; Mullick et al., 2015; Verstraeten et al., 2016). Studies with stroke survivors have shown differential patterns of motor outcomes depending on the cognitive deficits of patients (Čengić et al., 2011; Påhlman et al., 2011). Moreover, repeated performance of a movement may not lead to meaningful improvement unless the task is performed within the functional demands of a relevant environment (Levin et al., 2014). In fact, the practice of manipulations that require more cognitive effort were already predicted to be more effective for motor learning compared to those that require less cognitive effort (Hochstenbach et al., 1998). In this endeavor, it is important to investigate the learning potential of patients with post-stroke cognitive and motor impairments by developing new therapeutic strategies that merge cognitive and motor intensive training.

### Virtual Reality as a Tool for Combined Cognitive and Motor Rehabilitation

Virtual reality (VR) can nowadays be seen as a valuable approach in stroke rehabilitation, particularly in the motor domain where studies showed benefits at the level of upper limb function and ADL (Laver et al., 2017). This is potentially related to the fact that VR allows creating conditions to optimize motor learning by promoting meaningful and iterative practice, together with the delivery of immediate feedback (Levin et al., 2014). Although less explored, VR also provides the opportunity to integrate the practice of cognitive and/or motor activities in more ecologically valid contexts (Rand et al., 2009; Faria et al., 2016a; Adams et al., 2018). In such scenarios, motor training could be combined with the execution of cognitive rehabilitation tasks consisting of activities for improving cognitive domains such as attention, memory, or executive functions. Moreover, limitations in cognitive function have been shown to have an effect on VR performance (Kizony et al., 2004), and thus VR systems should be designed to address different cognitive profiles. Although the evidence is still modest, some studies with VR for simultaneous motor and cognitive rehabilitation have shown the potential of such strategy (Rand et al., 2009; Kim et al., 2011; Lee et al., 2015; Cameirão et al., 2017). Hence, we argue that novel VR tools should focus on integrative cognitive and motor rehabilitation based on tasks that pose both cognitive and motor demands. Assuming the interdependence between the recovery processes, we may provide a more effective rehabilitation tool.

Here we present the results of a feasibility study with the Reh@Task, a multi-purpose desktop based virtual scenario that combines arm reaching and cognitive training through virtual adaptations for the training of memory and attention of traditional paper-and-pencil tasks.

### Previous Work With the Reh@Task

The Reh@Task is a multi-purpose VR scenario for upper limb reaching and cognitive training that has been deployed in different configurations and with different rehabilitation paradigms. It allows the customization of stimuli, training task and training progression. In its first version, it originated as an adaptation in VR of the Toulouse Piéron (TP) cancelation task for the training of attention (Faria et al., 2014). The prototype was our first attempt to combine motor and cognitive training. It was primarily an attention only task that consisted on selecting target elements from a pool of distractors through arm reaching. This concept was tested in a 1-month intervention case study with three stroke survivors that presented both motor and cognitive deficits. Results indicated improvements both at motor and cognitive levels, suggesting the feasibility of the proposed approach (Faria et al., 2014). Following those results, the Reh@Task prototype was proposed with stimuli customization – to encompass varying cancelation tests with different stimuli – and the incorporation of a memory variant of the cancelation task for the training of memory, always relying on upper limb reaching movements. Thus, this new prototype enables the simultaneous training of upper limb reaching movements, memory, and attention. One of the advantages of a system such as the Reh@Task is that it can be easily customized to test different research hypotheses on the impact of such technology on stroke survivors with different profiles. In a previous controlled impact study, the Reh@Task was used to evaluate if cognitive tasks supported by personalized stimuli with positively valence could lead to improved motor and/or cognitive outcomes in an understudied population in comparison with standard rehabilitation. This was done through stimulus selection from emotionally tagged pictures and through content personalization to patients’ preferences, including music, in a group of sub-acute stroke survivors with mild cognitive impairment (MCI) (Cameirão et al., 2017). Results showed that the Reh@Task was as effective as standard rehabilitation, although motor and cognitive improvements were poor in both groups. This suggested that patients with MCI have a poorer recovery prognostic, specifically when presenting simultaneous motor and cognitive deficits. In fact, there is evidence that cognitive deficits interfere with motor recovery (Mullick et al., 2015), and that patients with MCI might have more difficulties in dual-tasking (Schaefer and Schumacher, 2010).

In the present study, the Reh@Task was used with stimuli different to those used in the above mentioned studies, focusing on neutral stimuli that do not have an emotional charge and are traditionally used in standard rehabilitation (symbols, numbers, and letters), with a difficulty progression based on computational models of how stimuli properties affect task difficulty (Faria and Bermúdez i Badia, 2015). Further, in this case our population is chronic. Hence, this study presents a novel cognitive training, task progression, tested on a different patient population, and compares the impact of such approach to time matched conventional rehabilitation activities. We hypothesize that rehabilitation with the Reh@Task will result in improved motor and cognitive outcomes when compared to patients in the standard rehabilitation condition.

## Materials and Methods

### Experimental Setup and Reh@Task

**FIGURE 1:**
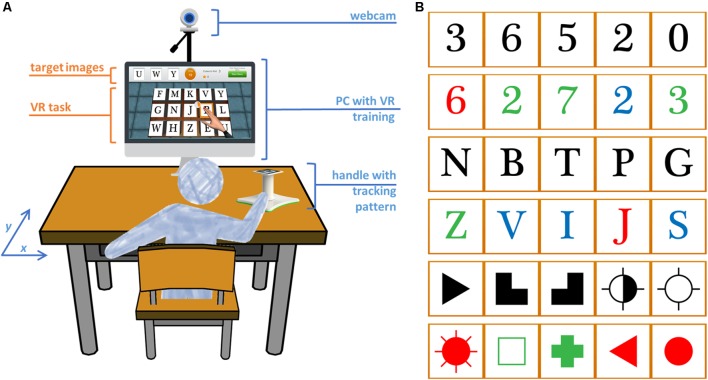
Experimental setup and VR task. **(A)** The user works on a tabletop and arm movements are captured by augmented reality pattern tracking. These movements are mapped onto the movements of a virtual arm on the screen for the execution of the cancelation task. **(B)** The target stimuli can be letters, numbers, and symbols in black or different colors. The target stimuli in this picture are ordered by increasing complexity.

The setup consists on a PC (OS: Windows 7, CPU: Intel core 2 duo E8235 at 2.80 GHz, RAM: 4 Gb, Graphics: ATI mobility Radeon HD 2600 XT), a PlayStation Eye camera (Sony Computer Entertainment Inc., Tokyo, Japan) and a customized handle with a tracking pattern. The user works on a tabletop, facing a LCD monitor (24″) and moves the handle on the surface of the table with his/her paretic arm (**Figure 1A**). 2D upper limb reaching movements are captured through a camera-based Augmented Reality (AR) pattern tracking software (AnTS)^1^ (Mathews et al., 2007). For adapting the task to individual users, the VR scenario has a built-in calibration function that normalizes the motor effort required in the task to the skillset of the user. The movements of the user are then mapped onto the movements of a virtual arm on the VR environment.

The Reh@Task is based on traditional cancelation tests for the training of attention, and has been extended to incorporate numbers, letters and symbols, and the training of memory, and progressive difficulty adjustment according to the evolution of the patient (**Figure 1B**). The task consists on finding target elements within a pool of distractors. In the memory variant, the targets need to be memorized first and are hidden during target selection. The VR cancelation task has incremental difficulty and is adjusted to the individual performance of each user. There is a total of 120 difficulty levels that were defined through a participatory design study, where the input of 20 health professionals was operationalized in quantitative guidelines (Faria and Bermúdez i Badia, 2015). The progression of difficulty is made through the manipulation of the number of targets and distractors, the type of stimulus, the time available to solve the task, the time for selection and, in the memory variant of the task, the amount of time for memorizing the target. These parameters are all operationalized in a way that increases the difficulty of the task incrementally (see Faria et al., 2016b) for further details on the difficulty adjustment algorithm). In summary, for higher difficulty levels, more target and distractor elements appear, less time is available for completing the task and memorizing the target images, and action selection is quicker. When a patient does not solve a specific level in the established timing, more time is given for that level. This additional time can be incremented up to three times. If the user fails three times in a row, he/she goes back to the previous level. If the user succeeds, the level must then be successfully performed within the original established time.

Finally, a rule was defined to select the starting level in each training session according to:

$${StartLevel}_{t}={StartLevel}_{t-1}+\left({EndLevel}_{t-1}-{StartLevel}_{t-1}\right)/2$$

where StartLevel and EndLevel denote the starting and finishing levels, respectively, and t indicates the session number. For instance, if the level achieved by a participant in the first session was 28, the second session would start in level 14 (28/2). If in the second session level 44 would be reached, the third session would start in level 29 [14 + (44 - 14)/2], and so on for the following levels.

### Participants

**FIGURE 2:**
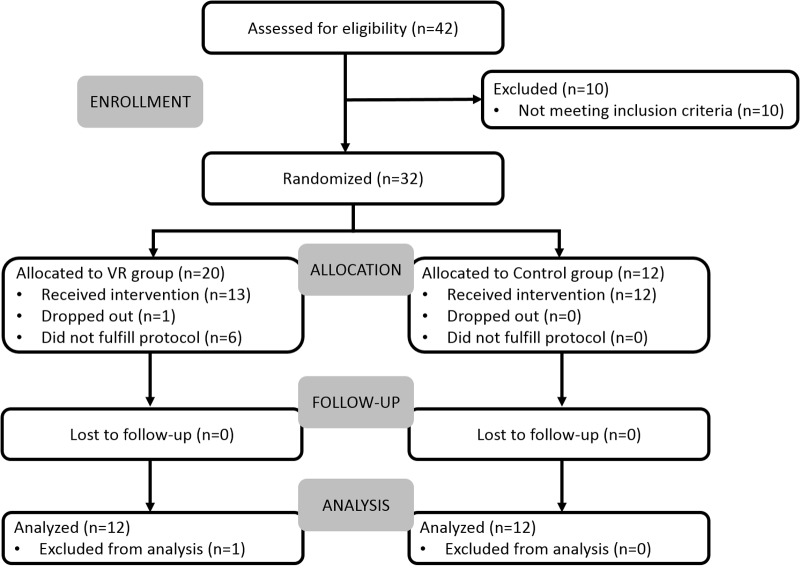
Flow diagram of enrollment, intervention allocation, follow-up, and data analysis.

The sample was a convenience sample with a final size of 24 participants recruited at two outpatient rehabilitation units of CMM – Centros Médicos e Reabilitação (Murtosa and Aveiro, Portugal) between June of 2015 and April of 2017. The inclusion criteria were the following: chronic stroke (>6 months); undergoing occupational therapy rehabilitation at CMM; motor impairment of the upper extremity with sufficient observable movement to perform the virtual task, corresponding to a minimum score of 28 in the Motricity Index (MI) (Demeurisse et al., 1980) for elbow flexion and shoulder abduction combined; cognitive deficit but with enough capacity to understand the task and follow instructions, as assessed by the therapists; and able to read and write. Exclusion criteria included: history of premorbid deficits; unilateral spatial neglect assessed through paper-and-pencil cancelation tests; severe depressive symptomatology with a score above 20 points in the Geriatric Depression Scale (GDS) (Yesavage et al., 1983); and vision disorders that could interfere with the execution of the task. Thirty-two stroke survivors were included and randomized for participation in this study. Minor deviations from inclusion/exclusion criteria were permitted for two participants, and did not affect the participants’ health, wellbeing, and rights (1 participant was 5 months post-stroke; 1 participant had a GSD score of 22). 25 participants completed the protocol, 1 dropped out, and 6 did not fulfill the experimental protocol. One participant was not included in the analysis because this participant was later confirmed to be in the acute stage of stroke (**Figure 2**). Hence, 24 participants (12 in VR group, 12 in Control group) were included in the analysis (**Table 1**). There were no significant differences between groups in demographics, except for age, the control group was significantly older (Mann–Whitney, *U* = 31.0, *p* = 0.017). This study was carried out in accordance with established ethical guidelines and was approved by the board of CMM – Centros Médicos e Reabilitação. All participants gave written informed consent in accordance with the Declaration of Helsinki.

**Table 1:** Characteristics of participants.

Group	Sex	Age	Schooling	Months post-stroke	Type of stroke	Side of lesion	GDS
**VR**	F	59	4	55	I	L	11
	M	57	6	40	I	L	8
	M	57	4	70	I	R	6
	F	55	6	16	I	L	8
	M	58	9	7	I	L	12
	M	78	4	6	I	R	14
	M	64	7	15	I	L	16
	F	68	3	14	I	L	22
	M	61	4	13	I	R	3
	M	37	9	23	H	L	4
	F	41	4	10	I	L	18
	M	51	12	30	I	R	3
**Control**	M	67	3	30	I	R	17
	F	76	4	61	I	L	17
	M	85	4	34	I	L	8
	M	75	4	84	I	L	11
	F	75	3	132	I	R	16
	M	65	4	12	I	L	13
	M	80	4	9	I	R	8
	F	62	3	88	I	R	14
	M	54	4	5	I	R	0
	F	53	11	18	U	L	10
	M	70	17	9	H	L	7
	F	65	7	12	U	L	13
**VR**	**4/8**	**57.1 ± 11.0**	**6.0 ± 2.8**	**24.9 ± 20.3**	**1/11/0**	**8/4**	**11.2 ± 5.7**
**Control**	**5/7**	**68.9 ± 9.8**	**5.7 ± 4.2**	**41.1 ± 41.0**	**1/9/2**	**7/5**	**11.2 ± 5.0**


**Sex: F, female; M, male; Schooling is presented in years; Type of stroke: I, ischemic; H, hemorrhagic; U, unknown; Side of lesion: L, left; R, right.**

### Experimental Protocol

This study followed a between-subjects design. After recruitment and baseline assessment, the participants were randomly assigned to one of two groups (VR or Control) by a researcher not involved in data collection, using the Research Randomizer, a free web-based service that offers instant random sampling and random assignment (Research Randomizer^2^). Participants in the VR group underwent 12 sessions of 45 min with the Reh@Task, three times a week, for 1 month. Before the first session, participants went through an average of three short training trials with the Reh@Task with TP abstract stimuli. The training was intended to provide a clear understanding of the VR task, as well as to become used to the natural user interface (AnTS). After assuring that the patient understood the task and interface instructions, the intervention started with the attention variant of the task, then switched to memory, and so on intermittently. The control group intervention was time-matched and included twelve sessions of 45 min of standard occupational therapy, spatial and time orientation activities, and writing training. Both interventions were in addition to conventional occupational therapy that typically entails 2–3 weekly sessions of 45–60 min and includes upper limb motricity training, practice of fine motor skills, cognitive-motor training, dexterity training, ADL, normalization of muscle tone, balance training and communication training. Participants underwent motor and cognitive assessment through a number of standardized clinical scales, at baseline, end of treatment and 1-month follow-up.

### Cognitive, Motor, and Functional Assessment

Cognitive and motor scales that are widely applied clinically and in research were used to determine impairment severity and to measure cognitive and motor recovery. The assessor was not blind for the type of intervention. The cognitive profiling was made through the Montreal Cognitive Assessment (MoCA) (Freitas et al., 2011), which provides sub-scores for the following domains: Executive Functions, Naming, Memory, Attention, Language, Abstraction, and Orientation. The attention task-related capabilities were assessed with the Single Letter Cancelation (SLC) (Diller et al., 1974), the Digit Cancelation (DC) (Mohs et al., 1997) and the Bells Test (BT) (Gauthier et al., 1989). Motor deficits were assessed through the upper extremities part of the Fugl-Meyer Assessment Test (FM-UE) (Fugl-Meyer et al., 1975) for motor and joint functioning of the paretic upper extremity. Of the total score of 66, we also analyzed separately proximal (shoulder, elbow, forearm, coordination, 42/66) and distal (wrist, hand, 24/66) function. For functionality of the paretic upper extremity, the Chedoke Arm and Hand Activity Inventory (Barreca et al., 2004) (CAHAI) was used. MI was used to assess muscle power of the paretic upper extremity. Spasticity was assessed through the Modified Ashworth Scale (MAS) (Bohannon and Smith, 1987). Finally, the Barthel Index (BI) (Mahoney and Barthel, 1965) was used to assess independence in activities of daily living (ADLs).

### Data Analysis

The normality of distributions was assessed using the Kolmogorov–Smirnov test for normality. Because most distributions deviated from normality, non-parametric statistical tests were used. Hence, central tendency and dispersion measures of the variables are presented as median and interquartile range (IQR), respectively. For improvements in clinical scores, we show the mean and standard deviation (SD) for an easier comparison with the literature. Differences between groups in demographic and clinical data at baseline were assessed using a Mann–Whitney *U* test in interval and ordinal variables, and a Pearson’s chi-square (χ^2^) test in nominal variables. A per-protocol analysis was used. For within-group changes over time across the three evaluation moments (baseline, end of treatment, and follow-up), a Friedman test for related samples was used and reported as χ^2^ (degrees of freedom). The Wilcoxon’s T matched pairs signed ranks (one-tailed because we predicted improvement over time in both groups) was used for further related pairwise comparisons with respect to baseline. No correction was applied to account for the number of pairwise comparisons, as non-parametric tests are already considered conservative. To compare groups at the end of treatment and follow-up, for each group we computed the improvement with respect to baseline. We used a one-tailed Mann–Whiney *U* test to test the hypothesis that improvements in the VR group were superior against the control group.

The Reh@Task software logged data on patient task performance (errors, number of targets and distractors, type of stimuli, time to completion) as well as the movement traces of the paretic arm, smoothed using a Gaussian window of 1 second. Performance improvements over time in the VR group were assessed by comparing the performances of each patient at the first and last training sessions. The error rates were computed as a percentage for each type of stimulus during the 12 training sessions. Movement smoothness was computed from the movement traces by counting the number of movement sequences, defined as trajectory segments in-between null acceleration points. To assess improvements in range of movement (ROM) over time, changes in the tracked position of the hand were assumed in the *x*- and *y*-axis of the tabletop surface, and the average improvements of the last three sessions were compared against the average of the 3 first sessions. All comparisons were performed using the two-tailed Wilcoxon’s *T* matched pairs signed ranks test.

Effect sizes (*r*) are reported on the pairwise comparisons and are computed as *Z*/√*N* (Rosenthal, 1991). The criteria for interpretation of the effect is 0.1 = small, 0.3 = medium, and 0.5 = large. For all statistical tests, a significance level of 5% (α = 0.05) was set. Data were analyzed using Matlab (MathWorks Inc., Natick, MA, United States) and IBM SPSS Statistics for Windows, Version 22.0 (Armonk, NY, United States: IBM Corp).

## Results

### How Effective Is Cognitive Training With Reh@Task as Compared to Conventional Rehabilitation?

The baseline MoCA total scores were balanced between groups (*U* = 60.5, *p* = 0.503, *r* = 0.18), and so were the scores in MoCA subdomains (data not shown). Also balanced were the number of errors in SLC (*U* = 64.5, *p* = 0.659, *r* = 0.09), DC (*U* = 57.5, *p* = 0383, *r* = 0.19), and BT (*U* = 58.5, *p* = 0.431, *r* = 0.16).

The analysis of the scores over time for each group, considering the three evaluation moments (baseline, end of treatment, and follow-up), showed a significant impact on MoCA total score and some of its subdomains in both groups (**Table 2**). Specifically, the VR group displayed a significant effect in MoCA-Total [χ^2^(2) = 8.3, *p* = 0.016], MoCA-Recall [χ^2^(2) = 6.2, *p* = 0.046], and MoCA-Orientation [χ^2^(2) = 8.4, *p* = 0.015]. The control group showed a significant effect in MoCA-Total [χ^2^(2) = 9.1, *p* = 0.010], MoCA-Language [χ^2^(2) = 6.1, *p* = 0.047], and MoCA-Recall [χ^2^(2) = 6.1, *p* = 0.048]. Further pairwise comparisons with respect to baseline indicated that for the MoCA total score, both groups showed a significant improvement at end of treatment [VR: *T* = 12.5, *Z* = 1.83, *p* = 0.034, *r* = 0.37; Control: *T* = 3.0, *Z* = 2.68, *p* = 0.003, *r* = 0.55], and follow-up [VR: *T* = 2.0, *Z* = 2.62, *p* = 0.004, *r* = 0.53; Control: *T* = 2.0, *Z* = 2.77, *p* = 0.003, *r* = 0.56]. Mean improvements in MoCA total score at end of treatment were 2.6 ± 4.3 in VR against 3.1 ± 2.8 in Control, and for follow-up 3.4 ± 3.5 in VR against 3.0 ± 3.0 in Control. For MoCA subdomains with significant effects over time, improvements were also significant at end of treatment and follow-up for both groups. For the cancelation tests, the VR group showed a significant effect over time for BT [χ^2^(2) = 6.6, *p* = 0.037] only. Pairwise comparisons with respect to baseline revealed that this effect comes from a significant improvement at follow-up (*T* = 2.5, *Z* = 2.40, *p* = 0.016, *r* = 0.49), but not at the end of treatment. The control group showed a significant effect over time for the DC [χ^2^(2) = 11.3, *p* = 0.004] and BT [χ^2^(2) = 10.5, *p* = 0.005], with significant improvements at end of treatment and follow-up. No significant differences were found in the between-groups analysis, when comparing the significant improvements in the VR group with those of the control group at end of treatment and follow-up.

**Table 2:** Scores in cognitive assessment at baseline, end of treatment and follow-up for VR and control conditions.

Measure	Virtual reality (*N* = 12)				Control (*N* = 12)			
		
	Baseline	End	Follow-up	*p*	Baseline	End	Follow-up	*p*
**MoCA**								
Total (maximum = 30)	**22.5 (6)**	**25.0 (4)^∗^**	** 26.0 (4)^∗∗^**	**0.016**	**21.5 (5)**	** 24.0 (5)^∗∗^**	** 24.0 (3)^∗∗^**	**0.010**
Executive (maximum = 5)	3.5 (3)	4.0 (2)	4.5 (2)	0.066	2.0 (2)	2.5 (2)	3.0 (2)	0.102
Naming (maximum = 3)	2.5 (1)	3.0 (0)	3.0 (0)	0.062	3.0 (1)	3.0 (2)	3.0 (0)	0.210
Attention (maximum = 6)	5.5 (2)	6.0 (1)	6.0 (2)	0.204	5.0 (2)	5.5 (1)	5.0 (1)	0.131
Language (maximum = 3)	2.0 (1)	2.0 (0)	2.0 (1)	0.527	**2.0 (2)**	** 2.0 (1)^∗^**	** 2.0 (0)^∗^**	**0.047**
Abstraction (maximum = 2)	2.0 (1)	2.0 (0)	2.0 (0)	0.247	2.0 (1)	2.0 (0)	2.0 (0)	0.091
Recall (maximum = 5)	**2.0 (3)**	** 3.0 (2)^∗∗^**	** 3.0 (2)^∗^**	**0.046**	**2.0 (3)**	** 3.0 (1)^∗^**	** 3.0 (2)^∗^**	**0.048**
Orientation (max = 6)	**6.0 (2)**	** 6.0 (0)^∗^**	** 6.0 (0)^∗^**	**0.015**	6.0 (1)	6.0 (0)	6.0 (0)	0.368
**Cancelation tests**								
SLC – Errors	1.5 (4)	1.0 (3)	1.5 (4)	0.900	3.0 (6)	2.0 (5)	2.5 (6.0)	0.115
DC – Errors	0.5 (3)	0.0 (1)	0.0 (1)	0.531	**2.2 (2)**	** 0.0 (2)^∗∗^**	** 0.5 (1)^∗∗^**	**0.004**
BT – Errors	**4.0 (5)**	**3.0 (4)**	** 2.0 (2)^∗∗^**	**0.037**	**5.0 (4)**	** 2.0 (4)^∗∗^**	** 3.5 (4)^∗^**	**0.005**


**Scores are presented as Median (IQR); p*, p-value; Friedman test, bold indicates a significant effect (p < 0.05) over time; significant one-tailed pairwise comparison with respect to baseline are indicated with ^∗^p < 0.05, ^∗∗^p < 0.01, respectively.*

### How Effective Is Motor Training With Reh@Task as Compared to Conventional Rehabilitation?

On the scores in motor assessment scales at baseline, the groups were balanced in the CAHAI (*U* = 43.0, *p* = 0.093), BI (*U* = 56.5, *p* = 0.360), and MAS (*U* = 54.0, *p* = 0.281). However, the groups were not balanced in FM-UE (*U* = 28.5, *p* = 0.010) and MI (*U* = 33.0, *p* = 0.024), with the control group having significantly higher scores in these two scales.

The analysis of the scores over time for each group, considering the three evaluation moments, showed for both groups a significant impact on FM-UE [VR: χ^2^(2) = 12.1, *p* = 0.002; Control: χ^2^(2) = 11.1, *p* = 0.004], CAHAI [VR: χ^2^(2) = 7.5, *p* = 0.023; Control: χ^2^(2) = 11.3, *p* = 0.004], and MI [VR: χ^2^(2) = 12.0, *p* = 0.002; Control: χ^2^(2) = 11.3, *p* = 0.004] (**Table 3**). On the FM-UE arm and hand subparts, both groups showed significant improvements over time for the hand domain [VR: χ^2^(2) = 8.4, *p* = 0.015; Control: χ^2^(2) = 7.7, *p* = 0.021], but only the VR group improved significantly in the arm part [VR: χ^2^(2) = 11.1, *p* = 0.004; Control: χ^2^(2) = 4.7, *p* = 0.097]. The control group showed an additional significant effect in MAS [χ^2^(2) = 7.6, *p* = 0.022], indicating a decrease in spasticity. There was no significant effect over time for BI. Further pairwise comparisons with respect to baseline indicated that for the VR group improvements were significant at end of treatment and follow-up in FM-UE [End: *T* = 0.0, *Z* = 2.20, *p* = 0.014, *r* = 0.45; Follow-up: *T* = 0.0, *Z* = 2.37, *p* = 0.009, *r* = 0.48], FM-Arm [End: *T* = 0.0, *Z* = 2.21, *p* = 0.013, *r* = 0.45; Follow-up: *T* = 0.0, *Z* = 2.20, *p* = 0.014, *r* = 0.45], FM-Hand/wrist [End: *T* = 0.0, *Z* = 1.83, *p* = 0.034, *r* = 0.37; Follow-up: *T* = 0.0, *Z* = 2.03, *p* = 0.021, *r* = 0.41], CAHAI [End: *T* = 0.0, *Z* = 1.86, *p* = 0.031, *r* = 0.40; Follow-up: *T* = 0.0, *Z* = 1.89, *p* = 0.029, *r* = 0.39], and MI [End: *T* = 7.5, *Z* = 1.78, *p* = 0.037, *r* = 0.36; Follow-up: *T* = 1.0, *Z* = 2.85, *p* = 0.002, *r* = 0.58]. For FM-Arm, the improvement compared to the control group was significantly higher (*U* = 45.0, *p* = 0.031, *r* = 0.38) at end of treatment and marginally significant at follow-up (*U* = 48.0, *p* = 0.055, *r* = 0.33). The control group showed significant improvements at end of treatment and follow-up in FM-UE [End: *T* = 0.0, *Z* = 2.03, *p* = 0.021, *r* = 0.41; Follow-up: *T* = 0.0, *Z* = 2.38, *p* = 0.008, *r* = 0.49], FM-Hand/wrist [End: *T* = 1.0, *Z* = 1.75, *p* = 0.040, *r* = 0.36; Follow-up: *T* = 0.0, *Z* = 2.21, *p* = 0.013, *r* = 0.45], CAHAI [End: *T* = 0.0, *Z* = 2.23, *p* = 0.013, *r* = 0.45; Follow-up: *T* = 0.0, *Z* = 2.21, *p* = 0.013, *r* = 0.45], and MI [End: *T* = 0.0, *Z* = 2.04, *p* = 0.020, *r* = 0.42; Follow-up: *T* = 0.0, *Z* = 2.38 *p* = 0.009, *r* = 0.48]. For the MAS, the improvements were only significant at follow-up [End: *T* = 0.0, *Z* = 1.41, *p* = 0.078, *r* = 0.29; Follow-up: *T* = 0.0, *Z* = 2.24, *p* = 0.012, *r* = 0.46], corresponding to a median decrease of one grade in this spasticity scale, specifically from 1+ to 1. Besides the significant difference in FM-Arm at end of treatment, no other significant differences were found in the between-groups analysis at end of treatment and follow-up.

**Table 3:** Scores in motor assessment at baseline, end of treatment and follow-up for VR and control conditions.

Measure	Virtual reality (*N* = 12)				Control (*N* = 12)			
		
	Baseline	End	Follow-up	*p*	Baseline	End	Follow-up	*p*
**FM-UE**								
Total (maximum = 66)	**28.0 (27)**	**32.0 (24)^∗^**	**33.0 (25)^∗∗^**	**0.002**	**45.5 (21)**	**51.0 (20)^∗^**	**51.0 (22)^∗∗^**	**0.004**
Arm (maximum = 42)	** 19.0 (15.5)**	** 23.5 (13.7)^∗^**	** 24.0 (13.5)^∗^**	**0.004**	31 (11.5)	32.5 (12.0)	32.5 (12.7)	0.097
Wrist/Hand (maximum = 24)	** 9.0 (10.7)**	** 9.0 (12.2)^∗^**	** 9.0 (12.2)^∗^**	**0.015**	**14.0 (9.5)**	**18.5 (8.7)^∗^**	**18.5 (9.5)^∗^**	**0.021**
**CAHAI** (maximum = 91)	**39.0 (40)**	**39.0 (38)^∗^**	**39.0 (38)^∗^**	**0.023**	**59.5 (33)**	**63.0 (30)^∗^**	**67.0 (27)^∗^**	**0.004**
**BI** (maximum = 100)	90.0 (23)	95.0 (25)	95.0 (25)	0.097	97.5 (44)	97.5 (44)	97.5 (44)	1.000
**MI** (maximum = 99)	**53.0 (31)**	**53.5 (20)^∗^**	**60.5 (25)^∗∗^**	**0.002**	**63.0 (21)**	**69.0 (16)^∗^**	**70.0 (15)^∗^**	**0.004**
**MAS** (maximum = 4)	1.5 (1.6)	1.5 (0.9)	1.5 (1.0)	0.504	** 1.5 (0.9)**	**1.5 (0.5)**	** 1.0 (0.5)^∗^**	**0.022**


**Scores are presented as Median (IQR); p*, p-value; Friedman test, bold indicates a significant effect (p < 0.05) over time; significant one-tailed pairwise comparison with respect to baseline are indicated with ^∗^p < 0.05, ^∗∗^p < 0.01, respectively.*

The mean improvements with respect to baseline at end of treatment and follow-up in the measures where a significant within-group effect over time was observed are presented in **Table 4**. For the VR and control groups, the observed average improvement in FM-UE was 4.6 ± 6.2 and 2.1 ± 3.6, respectively. This improvement in the VR group mainly comes from the FM-Arm subpart and strongly contrast with what was measured in the control group at end of treatment (3.7 ± 5.1 in VR against 0.8 ± 2.0 in Control, *p* = 0.031) and follow-up (4.0 ± 5.5 in VR against 0.9 ± 2.1 in Control, *p* = 0.055). The average improvements in the FM-Hand/wrist subpart, although being significant with respect to baseline, were modest for both groups at end of treatment (0.8 ± 1.4 in VR against 1.3 ± 2.3 in Control) and follow-up (0.9 ± 1.4 in VR against 1.8 ± 2.1 in Control). Also modest were the improvements in the CAHAI for both groups at end of treatment (0.8 ± 1.5 in VR against 2.7 ± 3.1 in Control) and follow-up (1.1 ± 1.8 in VR against 4.3 ± 4.9 in Control). These values are considerably below of what is considered a Minimal Detectable Change (MDC), which should be above 6.3 (Barreca et al., 2005). For the MI, the average improvements were higher in VR when compared to control at end of treatment (4.8 ± 8.3 in VR against 3.9 ± 5.4 in Control) and follow-up (9.1 ± 8.7 in VR against 5.3 ± 5.4 in Control), although not being significantly different.

**Table 4:** Mean improvement at end of treatment and follow-up.

Measure	End		Follow-up	
		
	VR	Control	VR	Control
FM-UE	4.6 ± 6.2	2.1 ± 3.6	4.9 ± 6.3	2.7 ± 3.6
FM-Arm	3.7 ± 5.1	0.8 ± 2.0	4.0 ± 5.5	0.9 ± 2.1
FM-Wrist/Hand	0.8 ± 1.4	1.3 ± 2.3	0.9 ± 1.4	1.8 ± 2.1
CAHAI	0.8 ± 1.5	2.7 ± 3.1	1.1 ± 1.8	4.3 ± 4.9
MI	4.8 ± 8.3	3.9 ± 5.4	9.1 ± 8.7	5.3 ± 5.4


**Improvements are presented as Mean ± SD.**

### Outcomes in Reh@Task Measures

#### Task Performance Measures

The Reh@task data allowed us to quantify the evolution of patients in the VR group over time in between assessment points. Several variables are considered for this analysis: difficulty level achieved during each training session, type of task (memory/attention), and type of stimulus.

**FIGURE 3:**
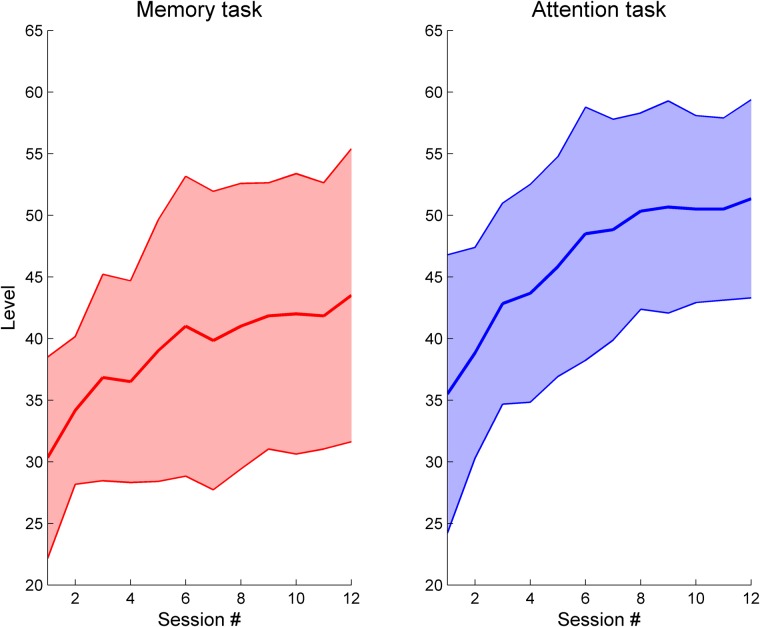
Task performance evolution over time in the Reh@Task. Data show the maximum difficulty level achieved per training session for the memory and attention tasks.

**FIGURE 4:**
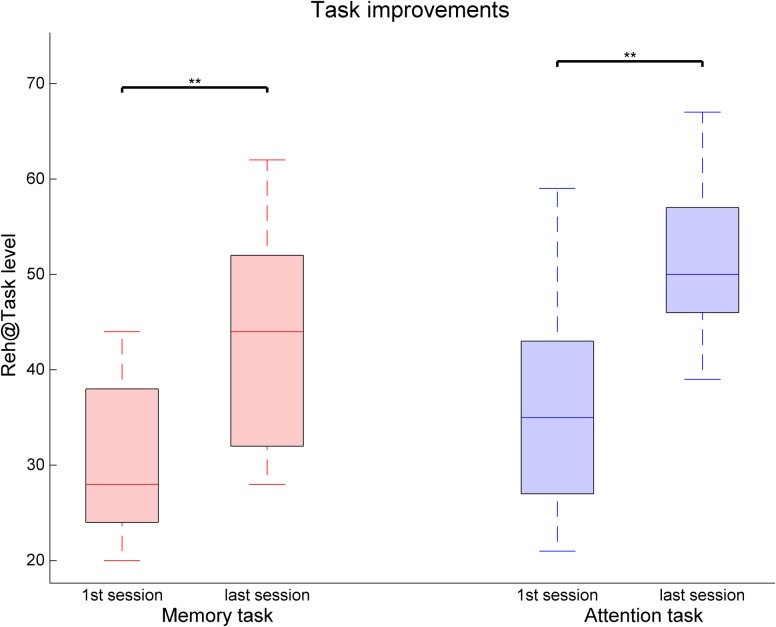
Task performance changes between the first and last training sessions for the memory and attention tasks in the Reh@Task. The whiskers indicate the most extreme data points that are not considered outliers. ^∗∗^ indicates *p* < 0.01.

When looking at changes over time, we observe that patients improve over time in both task types but display a deceleration as levels of higher difficulty are achieved (**Figure 3**). Patients achieve in average higher difficulty levels in the attention task, display a steeper slope, and exhibit a constant variability over time. In contrast, improvements in the memory task are slower, reaching lower difficulty levels and with increasing variability over time, indicating an uneven increased difficulty of this task in patients when compared to attention. Data show significant improvements in task performance between the first and last sessions [Attention: *Z* = 2.99, *p* = 0.003, *r* = 0.61; Memory: *Z* = 3.07, *p* = 0.002, *r* = 0.63] (**Figure 4**). There were comparable performances in the first session for both attention (*M* = 35.5 ± 11.3) and memory tasks (*M* = 30.3 ± 8.2), but the difference is statistically significant in the last training session [Attention: 51.3 ± 8.0, Memory: 43.5 ± 11.9, *Z* = 2.64, *p* = 0.008, *r* = 0.54].

**FIGURE 5:**
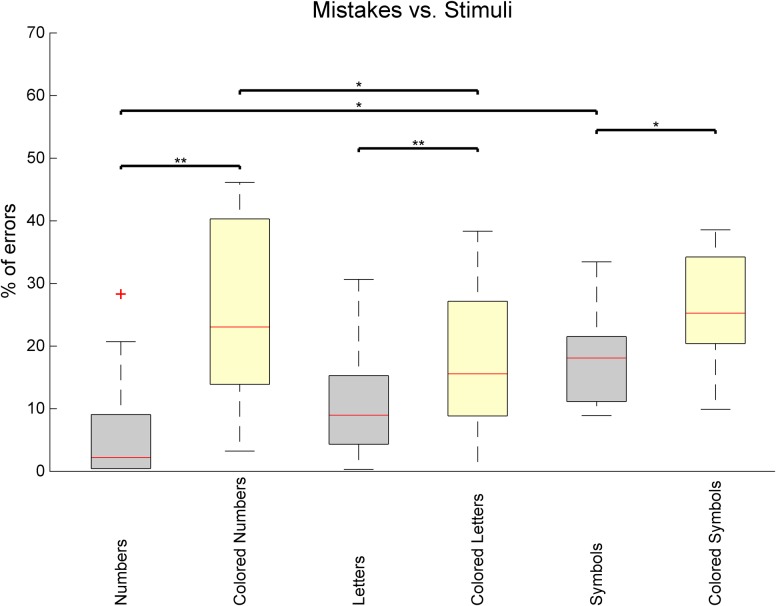
Percentage of task mistakes depending on the category of stimulus being presented in the Reh@Task. The whiskers indicate the most extreme data points that are not considered outliers. Outliers are represented as +. ^∗^ or ^∗∗^ indicates *p* < 0.05 or *p* < 0.01, respectively.

If task performance is analyzed by type of stimulus, distinct performances can be seen (**Figure 5**). An increasing average number of errors is observed for Numbers (6.5%), Letters (10.4%), and Symbols (17.5%), and the difference is significant when comparing symbols and numbers (*Z* = 2.12, *p* = 0.034, *r* = 0.43), showing a continuum of difficulty that is consistent with the level of abstraction of each category. In addition, all categories show a significantly increased error rate when comparing the black stimuli with their colored counterpart [Numbers: *Z* = 3.06, *p* = 0.002, *r* = 0.62; Letters: *Z* = 2.98, *p* = 0.003, *r* = 0.61; Symbols: *Z* = 2.43, *p* = 0.015, *r* = 0.50]. Interestingly, error rates are similar for colored numbers (25.50%) and for colored symbols (25.48%) despite numbers being easier than symbols when uncoupled with colors. Surprisingly, error rates are significantly lower for colored letters than for colored numbers [Colored Letters: 17.81%, Colored Numbers: 25.50%, *Z* = 2.12, *p* = 0.034, *r* = 0.43].

#### Motor Performance Measures

**FIGURE 6:**
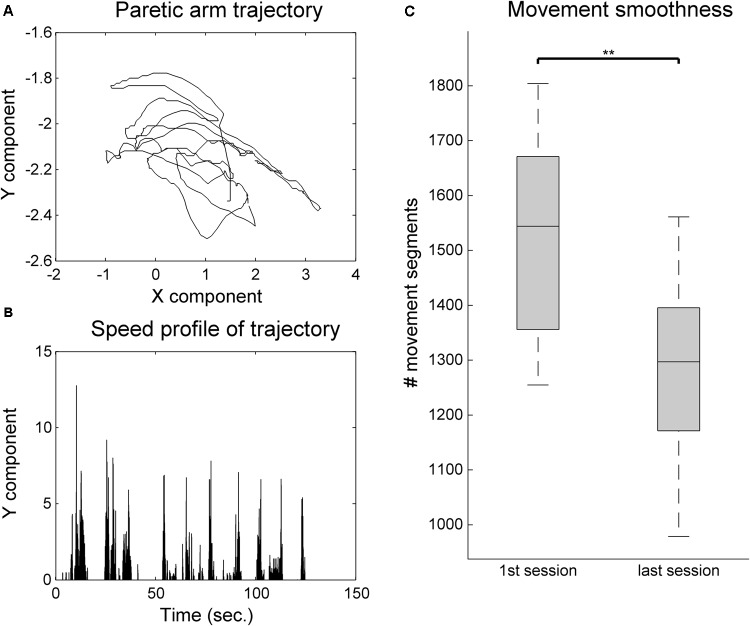
Movement smoothness analysis for the VR group. **(A)** Example 2-min sample of movement trajectory of one patient. **(B)** Computed speed profile of the sample in **(A)**. Movement sequence segments are identified in-between null acceleration points. **(C)** Movement smoothness changes between the first and last training sessions. The whiskers indicate the most extreme data points that are not considered outliers. ^∗∗^ indicates *p* < 0.01.

**FIGURE 7:**
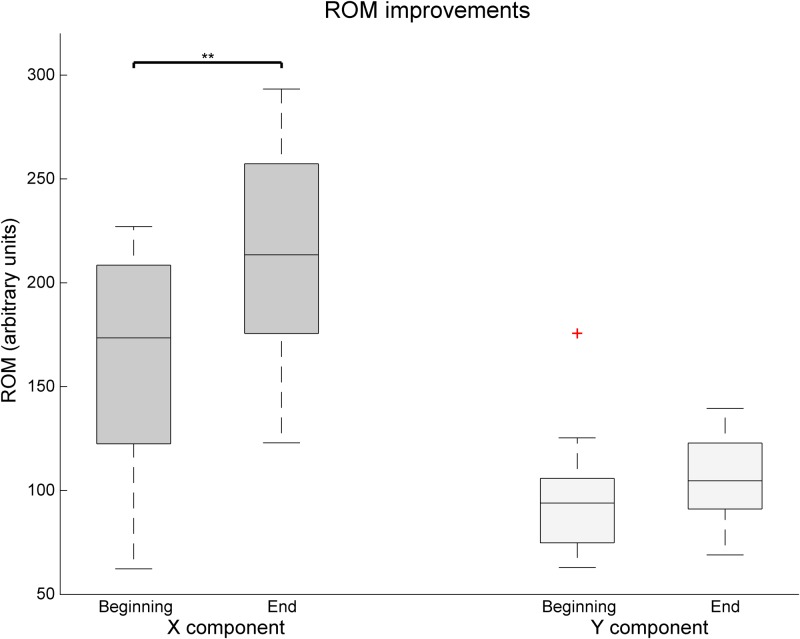
Changes over time of the *x* and *y* component of the Range of movement as assessed by the Reh@Task calibration. The whiskers indicate the most extreme data points that are not considered outliers. Outliers are represented as +. ^∗∗^ indicates *p* < 0.01.

The analysis arm movement trajectories provide information on both ROM and movement smoothness. The movement smoothness metric assumes that the movement trajectories that are built of less movement segments, that is, with less accelerations and decelerations, are indicative of a more controlled and smooth movement. A comparison of movement smoothness between the first and the last training sessions revealed a very significant decrease in the number of movement segments, indicating longer and smoother trajectories (*Z* = 2.93, *p* = 0.003, *r* = 0.60) (**Figure 6**). Finally, an analysis of the changes in ROM as assessed by the system’s calibration at the beginning of each session revealed significant improvements in the *x* (30.1% of improvement, *Z* = 2.67, *p* = 0.008, *r* = 0.54) component of the movement, but not on the *y* (**Figure 7**).

## Discussion

We presented a randomized controlled study with a VR cognitive and motor training task, the Reh@Task, consisting on a 1-month intervention with 24 chronic stroke survivors. We compared time-matched training with Reh@Task to standard occupational rehabilitation. During the intervention, all patients underwent conventional occupational therapy; only the VR group had specific training with the Reh@Task. The goal of this study was to investigate the benefits for stroke recovery of an integrative VR approach that combines cognitive and motor training. The main hypothesis behind this approach is that when approaching both motor and cognitive components, the context and situatedness of training impact its ecological validity. For this reason, both motor and cognitive challenges are personalized to each patient and presented as a single motor-cognitive VR task.

Our data show that both groups improved significantly in the motor domain in the FM-UE, CAHAI, and MI. However, in the total FM-UE the improvements in the VR group (4.6–4.9) were on average twice of those for the control group (2.1–2.7). This improvement in VR is superior to the ones observed in previous studies with similar VR paradigms in a chronic population (Cameirão et al., 2012; Maier et al., 2017). A more intensive (20 sessions in 1 month) motor-only intervention resulted on FM-UE improvements of about three points (Cameirão et al., 2012). A combined cognitive-motor approach, where the cognitive domain did not follow an automated adjustment approach but was more intensive (5 weekly sessions of 30 min during 6 weeks), led only to average improvements of less than 2 points in FM-UE (Maier et al., 2017). An analysis of our results in the FM components indicates that the improvement in the FM-Arm is significantly higher in comparison to control. Although both groups address proximal movements, this could be attributed to the nature of the VR task, which focuses on reaching movements. This is in line with other cognitive-motor studies with chronic stroke survivors where the training of hand motor competences in VR resulted in gains on manual abilities (Broeren et al., 2008). Nevertheless, our VR task does not address distal movements and comparable FM-Hand/Wrist improvements with the control group are achieved. These improvements in clinical scales are consistent with the Reh@Task data, that showed significant gains in ROM and movement smoothness. Concerning spasticity as measured by the MAS, we observed a significant reduction of one grade (from 1+ to 1) for the control but not the VR group. This is most likely related to the fact that the control group underwent more time of conventional occupational therapy, which includes normalization of muscle tone. Nevertheless, it has been argued that the 1+ and 1 grades do not have enough granularity do discriminate changes in spasticity (Pandyan et al., 1999).

Motor improvements did not generalize into clinically meaningful improvements in ADLs as measured by the BI and CAHAI. Considering that our sample is chronic and presents a very high BI and a low CAHAI at baseline, this indicates that these patients have high levels of independence despite their deficits. This suggests that effective strategies have been learned prior to the study that do not involve the paretic arm, leading to learned non-use, commonly observed in chronic populations (Wolf et al., 1989). If this is the case, an effective VR training should also incorporate strategies to address learned non-use (Ballester et al., 2016). This hypothesis is supported by previous results of an intervention with a modified version of the Reh@Task in a subacute population, in which improvements in CAHAI were larger, reaching meaningful values (Cameirão et al., 2017). This is also consistent with data from another integrative cognitive-motor VR study with patients in the 1st month post-stroke, where a mean improvement in BI of ∼20 points was registered (Kim et al., 2011), what strongly contrasts with the average 5 points improvement that we measured in our study with a chronic population.

The impact of both VR and control interventions in cognitive function was significant (3/30 in MoCA) but not different between groups. Still, our results strongly contrast with those obtained using a similar motor and cognitive training paradigm with chronic stroke where improvements in cognitive function where not significant after 6 weeks of training (Maier et al., 2017), despite being a more intensive training with five sessions a week. Both groups in our study showed improvements in total MoCA and recall, which suggests that both interventions had an impact in terms of general cognitive functioning and memory. VR showed an additional improvement in orientation, and the control group in language. The lack of improvements in other sub-domains could be explained by the fact that although MoCA has high sensitivity to detect post-stroke cognitive impairment (Godefroy et al., 2011), it is a screening tool and might have not fully detected the specific cognitive impact of this intervention. Both groups improved in attention as assessed by the cancelation tests. Hence, the VR group had improvements consistent with the dimensions trained in the Reh@Task, and consistent with the Reh@Task performance data. The performance data during VR training show significant improvements over time in both memory and attention training. The lower performance in the memory tasks is also consistent with the lower recall scores of MoCA at baseline. The analysis of task performance depending on the stimulus used supports the importance of the modeling effort of our personalization algorithm, which automatically adjusts the task configuration (including stimulus type, number of targets, and distractors) to provide an appropriate challenge to the patient.

A prototype version of the Reh@Task, combining attention and arm reaching only, was previously tested with three chronic stroke survivors in a less intensive intervention (Faria et al., 2014). In that pilot study, two patients showed improvements in motor and cognitive function, and in ADLs, indicating the potential of an approach that integrates motor and cognitive training. Later, a different customization of the Reh@Task was used in a controlled study with subacute stroke survivors (Cameirão et al., 2017). The intervention was time-matched to the one being presented here and contrasting results were obtained. In that case, the Reh@Task was configured to also train attention, memory and arm reaching, but pictures of positive valence were used instead. In terms of mean improvements, in the here presented study we observed higher improvements in total FM-UE (4.6–4.9 against 0.3–3.0) and MoCA (2.6–3.4 against -0.9–1.7), and lower improvements in CAHAI (0.8–1.1 against 6.6–11.1). These results are interesting because it would be expected to observe a higher impact of training in the subacute population, but this was not the case. The subacute population improved poorly in both motor and cognitive domains. A factor that could contribute to this result is the fact that the subacute population had higher cognitive deficits at baseline (median 20.0 against 22.5), and it has been suggested that cognitive functioning is associated with upper limb motor recovery (Mullick et al., 2015). Additionally, the subacute population had on average higher depressive symptomology (15.1 against 11.2) and less years of schooling (4.6 against 6.0). Both these factors have been associated with poorer cognitive performance (Zahodne et al., 2015; MacIntosh et al., 2017). However, the subacute population did better in the performance of ADLs as measured by the CAHAI. As previously mentioned these differences could be related to learned non-use that is often observed in chronic stroke patients, that limits the impact of actual rehabilitation gains (Wolf et al., 1989). This highlights the importance of an early use of rehabilitation strategies that prevent learned non-use.

We believe that the presented results are supportive of the viability of low-cost rehabilitation solutions that combine motor and cognitive training, such as the Reh@Task. These solutions show potential to be effective tools to address cognitive training in an integrative manner and can be easily deployed at home or at the clinic. Our data supports a larger impact in motor function than in cognitive function when compared to control. One possible reason could be the limited range of cognitive tasks implemented in Reh@Task that do not encompass all domains needed to be addressed in a comprehensive rehabilitation program. A second reason could be the limited ecological validity of the training tasks. Despite being integrative motor-cognitive tasks, these are still far from actual motor-cognitive tasks performed in ADLs. Previous work using VR cognitive training of ADLs in simulated environments like a virtual mall or a virtual city showed translation of competences to real world ADLs (Rand et al., 2009) and improved outcomes when compared to standard cognitive rehabilitation (Faria et al., 2016a). The relevance of such approaches can also be seen in a recent study with chronic stroke survivors that used a VR scenario for motor training based on the execution of virtual ADLs (Adams et al., 2018). After 8 weeks of treatment, a group of 15 patients showed a mean improvement of ∼6 points in FM-UE, which is superior to what we have observed in our study.

Although further research in this area is essential, this work presents a valuable step toward designing more effective rehabilitation technologies that combine motor and cognitive training relying on VR. In fact, the recent Cochrane review on the effect of VR in stroke rehabilitation reports that there are not enough studies to assess the impact of VR in cognitive function (Laver et al., 2017). Hence, we believe that our contribution is relevant to the field. Nevertheless, this study has some limitations that should be considered. First, due to sequential admittance into the study, we used a completely randomized design, resulting in a heterogeneity of groups in age and FM baseline measures. The fact that groups differ in FM may also imply different recovery profiles. Second, although the use of standard of care as control is necessary, this control did not train the exact same competences as the Reh@Task. Third, the use of screening instruments for the assessment of the improvements in cognitive function in this context may lack the sensitivity to capture small improvements in the different domains addressed.

## Author Contributions

AF, MC, and SB defined and designed the research study, analyzed the data, and interpreted the results. JC, JA, and GC ran the intervention and collected the data. All authors revised and approved the current version of the manuscript.

## Conflict of Interest Statement

The authors declare that the research was conducted in the absence of any commercial or financial relationships that could be construed as a potential conflict of interest.

## Footnotes

1 http://neurorehabilitation.m-iti.org/tools/ants

2 https://www.randomizer.org/
